# *In-situ* construction of Zr-based metal-organic framework core-shell heterostructure for photocatalytic degradation of organic pollutants

**DOI:** 10.3389/fchem.2022.1102920

**Published:** 2023-01-04

**Authors:** Yasmeen S. Abdel Aziz, Moustafa M. S. Sanad, Reda M. Abdelhameed, Ayman H. Zaki

**Affiliations:** ^1^ National Institute of Oceanography and Fisheries (NIOF), Cairo, Egypt; ^2^ Central Metallurgical Research and Development Institute, (CMRDI), Cairo, Egypt; ^3^ Applied Organic Chemistry Department, Chemical Industries Research Institute, National Research Centre, Giza, Egypt; ^4^ Materials Science and Nanotechnology Department, Faculty of Postgraduate Studies for Advanced Sciences, Beni-Suef University, Beni Suef, Egypt; ^5^ International Center for Materials Nanoarchitechtonics (WPI-MANA), National Institute for Materials Science, Tsukuba, Japan

**Keywords:** photocatalysis, MOFs, ferrite, core-shell, visible light, dyes

## Abstract

Photocatalysis is an eco-friendly promising approach to the degradation of textile dyes. The majority of reported studies involved remediation of dyes with an initial concentration ≤50 mg/L, which was away from the existing values in textile wastewater. Herein, a simple solvothermal route was utilized to synthesize CoFe_2_O_4_@UiO-66 core-shell heterojunction photocatalyst for the first time. The photocatalytic performance of the as-synthesized catalysts was assessed through the photodegradation of methylene blue (MB) and methyl orange (MO) dyes at an initial concentration (100 mg/L). Under simulated solar irradiation, improved photocatalytic performance was accomplished by as-obtained CoFe_2_O_4_@UiO-66 heterojunction compared to bare UiO-66 and CoFe_2_O_4_. The overall removal efficiency of dyes (100 mg/L) over CoFe_2_O_4_@UiO-66 (50 mg/L) reached >60% within 180 min. The optical and photoelectrochemical measurements showed an enhanced visible light absorption capacity as well as effective interfacial charge separation and transfer over CoFe_2_O_4_@UiO-66, emphasizing the successful construction of heterojunction. The degradation mechanism was further explored, which revealed the contribution of holes (h^+^), superoxide (•O_2_^−^), and hydroxyl (•OH) radicals in the degradation process, however, h^+^ were the predominant reactive species. This work might open up new insights for designing MOF-based core-shell heterostructured photocatalysts for the remediation of industrial organic pollutants.

## 1 Introduction

Among various industrial sectors, the textile industry takes prominence due to the high utilization of water, raw materials, and chemicals including acids, chelating and bleaching agents, dyes, surfactants, *etc.* As a consequence, tremendous volumes of wastewater are released from this industry. It is estimated that 20% of global industrial wastewater emerges merely from textile industries (Holkar et al., 2016). In general, the textile effluent is characterized by high pH, intense color, high chemical and biochemical oxygen demands (COD and BOD5, respectively), and high concentrations of total suspended and dissolved solids (Yaseen and Scholz, 2019). Nevertheless, the composition of such effluents varies considerably in concentration and toxicity depending on the utilized chemicals, operating conditions, and the employed manufacturing steps (Ramos et al., 2021). Approximately 700,000 tons of synthetic dyes are produced annually and around 30% of this dyestuff ends up as industrial effluent (Al-Mamun et al., 2019). Owing to their complex aromatic structure and non-biodegradable nature, most of these dyes may present carcinogenic and/or mutagenic potentials to human health and aquatic ecosystem (Nidheesh et al., 2013; Dihom et al., 2022). Hence, efficient treatment of textile wastewater before discharge into water bodies has become of crucial importance.

Inspired by the natural photosynthesis process, photocatalysis has currently emerged as a promising green approach for the conversion of solar energy into chemical energy (Gao et al., 2017). Due to its high efficiency, feasibility, low energy consumption, and eco-friendly feature, semiconductor-induced photocatalysis has been successfully harnessed in diverse applications including energy storage and conversion (Wei et al., 2021; Han et al., 2022; Qin et al., 2022), CO_2_ reduction (Li et al., 2020; Xiong et al., 2020), organic synthesis (Zhang et al., 2019b; Xiong and Tang, 2021), Cr(VI) reduction (Yi et al., 2019; Zhang et al., 2020) and water treatment (Zeng et al., 2018; Feng et al., 2022; Shi et al., 2022). Up to present, several semiconductor photocatalysts have been intensively studied such as metal oxides [TiO_2_, ZnO, Fe_2_O_3_ (Ba-Abbad et al., 2013; Franking et al., 2013; Kreft et al., 2020)], metal sulphides [MoS_2_, CdS, In_2_S_4_ (Ning et al., 2019; Liang et al., 2021; Pan et al., 2021)], and organic semiconductors [(g-C_3_N_4_, perylene diimide, covalent organic framework (Zhou et al., 2018; Sivula, 2020; Zhou et al., 2021)]. Nevertheless, the photocatalytic performance of these catalysts is far unsatisfactory owing to various limitations like photocorrosion, low photon absorption efficiency, inefficient charge separation, most importantly; deficiency of effective and stable catalytic sites to maintain dynamic photocatalytic reactions (Gao et al., 2017).

As a distinct group of organic-inorganic hybrid crystalline porous materials, metal-organic frameworks (MOFs) have shown considerable potential in a variety of applications involving adsorption, drug delivery, gas storage and separation, and catalysis (Lei et al., 2018; Wang et al., 2020a; Wang et al., 2020b; Connolly et al., 2020; Younes et al., 2022). Due to their distinguished features such as tunable pore structure, high specific surface area with abundant catalytic active sites, and adjustable electronic and optical properties, MOFs have recently perceived unparalleled progress in the field of photocatalysis (Qin et al., 2020; Xia et al., 2021). Unlike conventional photocatalysts, MOFs are characterized by a special charge transition mechanism, where, the electronic states are localized, reducing the transmission distance of photoinduced carriers (Liang et al., 2019; Zhang et al., 2021c). Upon light illumination, the organic linkers, as light-absorbing antennas, and metal clusters, as semiconductor quantum dots, are excited to generate electron-hole pairs (Dey and Gogate, 2021). Consequently, several photo-excitation pathways are proposed to explore the photon harvesting process in MOF-based photocatalysts such as metal-to-ligand charge transfer (MLCT), metal-to-metal-to-ligand charge transfer (MMLCT), ligand-to-metal charge transfer (LMCT), and ligand-to-ligand charge transfer (LLCT) (Wen et al., 2019).

Beyond other reported MOF’s, zirconium Zr(IV)-based MOFs (e.g. UiO-66), have drawn tremendous interest because of their superb thermal and chemical stability even in acidic and some basic mediums, which is mostly attributed to the robust interaction between Zr-O clusters and carboxylate ligands (Yuan et al., 2018; Yuan et al., 2021). Hence, Zr-MOFs have emerged as an exciting class for photocatalytic potential applications in an aqueous environment (Zhang et al., 2021d; Zhang et al., 2021c). However, the photocatalytic performance of UiO-66(Zr) still does not reach the utmost level due to its relatively wide bandgap energy (∼3.8 eV). Thus, UiO-66 can only absorb light in the ultraviolet region (3–4%), leaving more than 90% of the solar spectrum unutilized. This in turn, results in a low photoconversion efficiency and limits the practical application of UiO-66 photocatalyst for solar light harvesting (Cheng et al., 2016; Gao et al., 2017). To fulfill the sustainable development concepts, several approaches have been embraced for promoting the photocatalytic efficiency of UiO-66(Zr) including bandgap engineering (Taddei et al., 2019), element doping (Qiu et al., 2019), ligand functionalization (Wang et al., 2021c), active site regulation (Shen et al., 2015b), *etc.* Interestingly, the construction of heterojunction structures has been reported as one of the most prospective strategies to boost the photocatalytic performance of Zr-MOFs through the formation of an interface between the two semiconductors (Subudhi et al., 2020; Zhang et al., 2021b). This intimate interfacial contact, in turn, favors accelerated charge transfer and boosts solar energy exploitation by modulating the band gap energy to attain the utmost photocatalytic efficiency (Jabbar and Graimed, 2022).

For example, Zhang et al. adopted facile adsorption and thermal conversion technique to encapsulate the *α*-Fe_2_O_3_ nanoclusters inside UiO-66 cavities for the construction of a visible light-driven *α*-Fe_2_O_3_@UiO-66 photocatalyst heterostructure for catalytic degradation of MB (Zhang et al., 2019a). Under visible light excitation, Fe_2_O_3_@UiO-66 displayed considerably boosted degradation performance. This prominent improvement of photoactivity of Fe_2_O_3_@UiO-66 could be explained by the synergetic interaction between UiO-66 and *α*-Fe_2_O_3_, which is beneficial to enhancing charge migration and lowering the recombination rate. Similarly, Yassin et al. prepared Ag_3_PO_4_/Zr-BDC/g-C_3_N_4_ ternary heterostructure for discoloration of MB under visible and solar irradiations (Yassin et al., 2022). Noteworthy, the UiO-66 bandgap energy is modulated from 3.72 eV to 2.91 eV in the Ag_3_PO_4_/Zr-BDC/g-C_3_N_4_ heterojunction, interpreting the effective absorption toward the visible spectrum. In comparison with pristine materials, Ag_3_PO_4_/Zr-BDC/g-C_3_N_4_ showed remarkably high degradation efficiency (95.0%) within 240 min under visible illumination, which might be credited to the spatial charge separation and prolonged carrier lifetime, confirmed by the significantly suppressed intensity of photoluminescence (PL) emission spectra. In addition, although numerous reports have revealed the splendid catalytic activity of Zr-MOF/metal oxide heterostructures for wastewater treatment, the high cost of some metal nanoparticles (e.g. noble metals), metal-ion leaching, and instability often restrict their practical applications (Zhang et al., 2021d; Mukherjee et al., 2022).

At present, cobalt ferrite (CoFe_2_O_4_), a spinel-type ferrite, has displayed marked potential as a versatile photocatalyst due to its facile synthesis, low cost, excellent magnetic anisotropy, high chemical stability, and narrow bandgap energy (∼2.0 eV) with visible light absorption capacity (Mathew and Juang, 2007; Mmelesi et al., 2021). In a recent study, it has been reported complete degradation of ciprofloxacin within 45 min of visible-light irradiation by CoFe_2_O_4_/ZnO nanoheterojunction (Shawky and Alshaikh, 2022). The excellent photocatalytic activity of the composite is explicated by the notable decline in the bandgap after the incorporation of CoFe_2_O_4_ as well as the inhibition of charge-transport resistance through the formed p-n nanoheterojunction. In another study, Khosroshahi et al. designed a novel magnetic CoFe_2_O_4_/Ce-UiO-66 nanocomposite through a self-assembly approach for photocatalytic oxidation of aliphatic alcohols. Upon visible irradiation, the embedded composite demonstrated superior performance for selective oxidation of alcohols with a conversion ratio of 75%–90% compared to 21% and 10% conversion for CoFe_2_O_4_ and Ce-UiO-66, respectively (Khosroshahi et al., 2021). Despite the fact that the magnetic behavior of CoFe_2_O_4_ has been extensively investigated, studies on its optical and photoelectrochemical properties are still in infancy, particularly, with concerns for poor efficiency owing to the swift recombination of carriers under light irradiation and its relatively low specific surface area (Kefeni and Mamba, 2020; Görmez et al., 2022). Based on that, the construction of core-shell Zr-MOF-based composites has been recognized as an attractive approach to effectively promote photostability and enlarge the specific surface area, which is conducive to exposing more active sites in the photocatalytic reaction (Liu et al., 2021).

In this report, we successfully prepared a novel CoFe_2_O_4_@UiO-66 core-shell heterostructure photocatalyst *via* a simple solvothermal route for photodegradation of textile MB and MO dyes under simulated solar irradiation. Even though the initial concentration of dyes in actual textile wastewater samples has been recorded as higher than 100 mg/L, the majority of reported studies involved dye removal with an initial concentration of less than 50 mg/L (Mukherjee et al., 2022). Thus, dyes of 100 mg/L as an initial concentration were used in this study to provide realistic conditions similar to that in real textile wastewater. The crystallinity, surface composition, morphology, porosity, thermal stability, and optical and photo electrochemical properties of the prepared catalysts were investigated in detail. In addition, radical quenching experiments were applied to explore the possible photocatalytic mechanism.

## 2 Experimental

### 2.1 Materials

Zirconyl chloride (ZrOCl_2_.8H_2_O, 99%), 1,4-benzenedicarboxylic acid (H_2_BDC) (C_8_H_6_O_4_, 98%), and benzoquinone (*p*-BQ) (C_6_H_4_O_2_, ≥98%) were purchased from Sigma-Aldrich cooperation. Ethylenediaminetetraacetic acid disodium salt (EDTA-2Na, C_10_H_14_N_2_Na_2_O_8_. H_2_O) and ammonium hydroxide (NH_4_OH) were supplied by BioChem Chemopharma Co. *N,N*-dimethylformamide (DMF, 99.5%), acetic acid (CH_3_COOH, ≥99%), and ethanol (C_2_H_6_O, ≥99.8%) were acquired from Carlo Erba Reagent Co, Ltd. 2-propanol (C_3_H_8_O, 99.7%) was provided by Merck. Methylene blue (MB, C_16_H_18_ClN_3_S) and methyl orange (MO, C_14_H_14_N_3_NaO_3_S) were supplied by LOBA Chemie Pvt. Ltd. Ferric chloride (FeCl_3_), cobalt (II) chloride hexahydrate (CoCl_2_.6H_2_O) were purchased from Oxford Lab Reagents Co. All reagents were of analytical grade and utilized without further purification. Deionized water was applied in the following experiments.

### 2.2 Preparation of photocatalysts

#### 2.2.1 Synthesis of CoFe_2_O_4_

CoFe_2_O_4_ nanoparticles were prepared by the co-precipitation method (Esmat et al., 2017). Briefly, 2.0 mol of FeCl_3_ and 1.0 mol of CoCl_2_.6H_2_O were dispersed into 30 ml of deionized water. Following that, NH_4_OH (1.0 M) was added dropwise until the pH reaches 10 and then left for complete precipitation. Afterward, the precipitate was collected *via* filtration, followed by washing it with deionized water. Subsequently, the precipitate was air-dried at 100°C in a drying chamber. Lastly, the dried powder was calcined in a muffle furnace for 2 h at 500°C to get CoFe_2_O_4_ nanoparticles.

#### 2.2.2 Synthesis of UiO-66

UiO-66 octahedrons were prepared through a modified scale-up procedure. Initially, 3.75 g of ZrOCl_2_ and 3.7 g of H_2_BDC were dispersed in 450 ml of DMF using ultrasonication for 60 min. Then, 20 ml of acetic acid was subsequently added to the mixture as a modulator to regulate the morphology of UiO-66. Next, the solution was poured into a 1,000 ml Teflon-lined stainless-steel reactor and heated for 24 h at 120°C. After cooling down, the white precipitate was obtained *via* filtration and washed meticulously several times with DMF and ethanol, respectively, to ensure the removal of any residual reactant. Finally, the UiO-66 nanoparticles were vacuum-dried for 12 h at 85°C.

#### 2.2.3 Synthesis of CoFe_2_O_4_@UiO-66 composite

**SCHEME 1:**
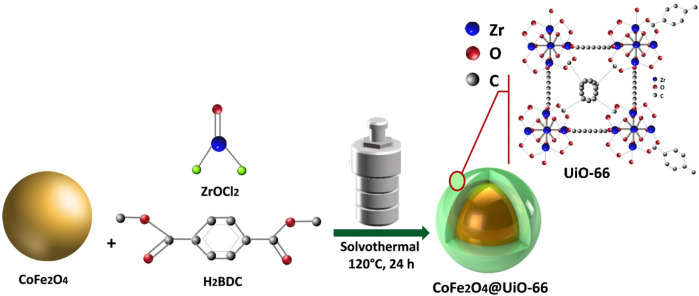
Schematic representation of CoFe_2_O_4_@UiO-66 composite preparation.

As illustrated in Scheme 1, a facile solvothermal method was adapted to prepare CoFe_2_O_4_@UiO-66 core-shell composite. Typically, 1.0 g of CoFe_2_O_4_ was dissolved in 450 ml DMF solution. Simultaneously, 3.75 g of ZrOCl_2_ and 3.7 g of H_2_BDC were dispersed in 20 ml of acetic acid. The prepared solutions were then mixed by ultrasonication for 60 min. Afterward, the homogenous solution was poured into a 1,000 ml Teflon-lined stainless-steel reactor and heated for 24 h at 120°C. After cooling down, the brown composite was separated and washed following the aforementioned washing process of UiO-66. In the end, the as-prepared product was vacuum-dried for 12 h at 85°C.

### 2.3 Characterization

The X-ray diffraction (XRD) patterns were recorded using an XRD diffractometer (PANalytical Empyrean, Switzerland) with Cu-Kα (ʎ = 1.5405 Å) radiation source, operating at a voltage and current of 30 mA and 40 kV, respectively. The morphology and microstructure characteristics of the as-fabricated materials were studied using a field emission scanning electron microscope equipped with an energy-dispersive spectrometer (EDS) system (FE-SEM, Zeiss Sigma 500 VP, Germany) and transmission electron microscope (TEM, JEOL JEM-2100F, Japan). Fourier transform infrared (FTIR) spectra were measured using a VERTEX 70 spectrophotometer (Bruker Optics, Germany). X-ray photoelectron spectroscopy (XPS) with Al-Kα radiation (Thermo ESCALAB 250XI, United State) was applied to examine the oxidation state of the prepared composite. N_2_ adsorption-desorption analysis was performed on BELSORP-MAX II surface area analyzer (MICROTRAC, Germany) at 77 K. Brunauer-Emmett-Teller (BET) and Barrett- Joyner-Halenda (BJH) methods were utilized to calculate the specific surface area and pore size distribution. Prior to measurement, the samples were vacuum-activated for 12 h at 150°C and then degassed for 3 h at 120°C. Thermogravimetric analysis (TGA) was conducted employing a LabSys EVO thermogravimetric analyzer (SETARAM, France) from room temperature to 800 °C under N_2_ atmosphere with a 10°C/min heating rate. The surface charge was measured on a Zeta potential analyzer (Zetasizer Nano ZS, Malvern, United Kingdom). The UV–visible diffuse reflectance spectra (UV–vis DRS) were determined using a UV–Vis spectrophotometer (Jasco V-770, Japan) with BaSO_4_ as a reference in the spectral range of 200–800 nm. The photoluminescence (PL) spectra were obtained by a fluorescence spectrometer (Jasco FP-6500, Japan) with an excitation wavelength of 320 nm for CoFe_2_O_4_ and 297 nm for UiO-66 and CoFe_2_O_4_@UiO-66.

### 2.4 Photoelectrochemical measurements

The photoelectrochemical characterization of the as-prepared catalysts was analyzed using a standard three-electrode electrochemical workstation system (Parastat 4,000 Princeton, United State) equipped with a Xenon lamp (150 W) as an irradiation source. Indium-tin-oxide (ITO) glass coated by the catalysts (ρ ∼ 30 Ω/cm^2^) served as the working electrode against the Pt sheet and saturated Ag/AgCl as the counter and reference electrodes, respectively. 0.1 M Na_2_SO_4_ was adopted as the electrolyte solution. The Mott-Schottky plots were estimated with the same electrochemical instrument at 500 Hz frequency under dark conditions. The electrochemical impedance spectroscopy (EIS) was recorded over a frequency range of 1 MHz–10 mHz with an amplitude of 50 mV at an open-circuit potential. Linear sweep voltammetry (LSV) tests were performed by sweeping the potential from 0 to 1.0 V.

### 2.5 Photocatalytic reaction

The photocatalytic performance of UiO-66, CoFe_2_O_4,_ and CoFe_2_O_4_@UiO-66 composite was assessed through the photodegradation of MB and MO dyes. The physicochemical properties of the former dyes are presented in Supplementary Table S1. In a typical procedure, 50 mg of catalysts were added to 25 mL of each dye solution (100 mg/L). After agitation for 60 min in dark to establish adsorption-desorption equilibrium, the solutions were irradiated by Solar Simulator (Oriel^®^Sol1A, Newport Co.) equipped with a 150 W xenon lamp (100 mW/cm^2^ light intensity). At regular time intervals, 100 µL of the sample solution was extracted, diluted to 700 µL with deionized water, and then centrifuged to separate the residual photocatalyst. The concentration of MB and MO was calibrated using a UV–vis spectrophotometer (UV-2600, Shimadzu, Japan) at maximum absorption wavelength (ʎ_max_) of 664 nm and 464 nm, respectively. The removal efficiency (%) was calculated following Eq. 1

$$Removal\;efficiency\;\left(\%\right)=\frac{C0-Ct}{C0}\times 100$$ (1)

where *C*_*0*_ and *C*_*t*_ (mg/L) are the dye concentration at initial and each interval time, respectively.

To investigate the photocatalytic mechanism, disodium ethylenediaminetetraacetic acid (EDTA-2Na), *p*-benzoquinone (BQ), and isopropanol (IPA) were used as trapping agents for photogenerated holes (h^+^), superoxide radicals (•O_2_^−^) and hydroxyl radicals (•OH), respectively. The concentration of the scavengers was set as 2 m and the photocatalytic assays were carried out following the same procedure described above under the same pH.

## 3 Results and discussions

### 3.1 Photocatalysts characterization

#### 3.1.1 Structure analysis

**FIGURE 1:**
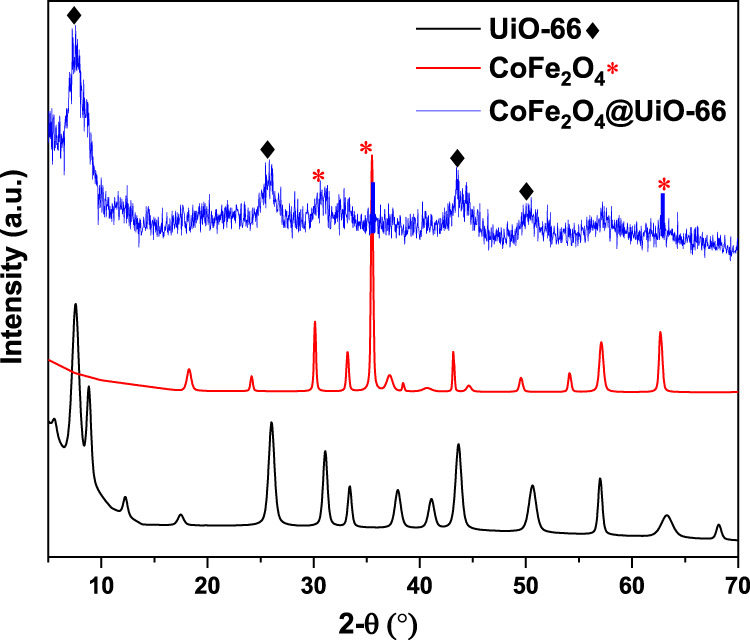
XRD patterns of UiO-66, CoFe_2_O_4_, and CoFe_2_O_4_@UiO-66 composite.

The crystallinity of the as-prepared samples was examined using XRD analysis. The simulated patterns of UiO-66 and CoFe_2_O_4_ are presented in Supplementary Figure S1. From Figure 1, it can be seen that the as-synthesized UiO-66 exhibited typical characteristic peaks cited at 7.5°, 8.6° indexed to the (111) and (200) crystal planes, respectively. Moreover, distinct peaks appeared at values of 12.21°, 17.4°, 25.9°, 31.0°, 33.4°, 37.7, 40.9°, 43.7°, 50.5°, and 57.1° agreed with (220), (400), (600), (711), (731), (751), (664), (933), (955), and (1242) crystal planes of UiO-66, respectively. This observation is in agreement with the former studies (Zhang et al., 2019c; Mirhosseini-Eshkevari et al., 2019; Gao et al., 2021), implying the successful preparation of the obtained material. Meanwhile, CoFe_2_O_4_ showed diffraction peaks at 18.27° (111), 30.21° (220), 35.51° (311), 43.32° (400), 53.9° (422), 57.14° (411), and 62.79° (440), which are consistent with the standard peak positions of spinel CoFe_2_O_4_ structure (Jia et al., 2019). For the CoFe_2_O_4_@UiO-66 heterojunction, the diffraction peaks correlating to UiO-66 appeared with a relative broadening and fluctuations of peak intensities, is indicative of a little alternation in the framework structural regularity (Bi et al., 2020). Even though the CoFe_2_O_4_ characteristic peaks noticeably weakened in the heterojunction owing to the *in‐situ* growth of the UiO-66 shell, they still could be distinguished. Given this, the aforementioned findings suggest the successful fabrication of the MOF-hybrid material.

**FIGURE 2:**
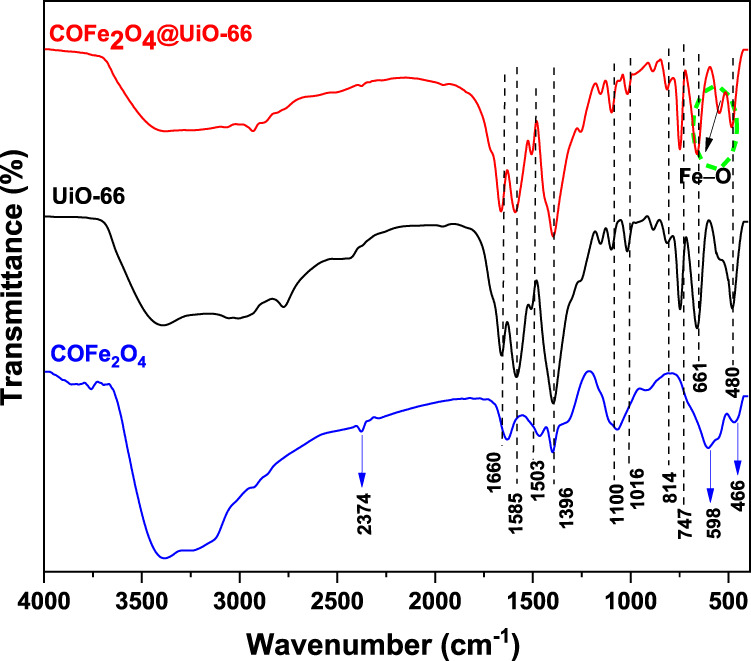
FTIR spectra of UiO-66, CoFe_2_O_4_, and CoFe_2_O_4_@UiO-66 composite.

FTIR spectroscopy was implemented to investigate the surface functionalization of the as-prepared samples. Figure 2 displays the FTIR spectra of UiO-66, CoFe_2_O_4,_ and CoFe_2_O_4_@UiO-66 composite. All samples showed a broad band at 3,200–3,500 cm^−1^ related to the O–H stretching vibration of absorbed water molecules (Ding et al., 2017; Basak et al., 2021). For UiO-66, typical bands can be identified at 1,585 and 1,396 cm^−1^, corresponding to O–C–O asymmetric and symmetric vibrations of the -COOH group of the BDC ligand, respectively (Liu et al., 2018). The weak vibrational bands at 1,503 and 1,660 cm^−1^ occurred by the C=C vibration of benzene ring (Shangkum et al., 2018) and the C=O carbonyl stretching in the BDC linker (Ebrahim and Bandosz, 2013), respectively. Meanwhile, the bands sited around 1,016 and 1,100 cm^−1^ are ascribed to the Zr–O stretching vibration of the framework (Chen et al., 2017; Bariki et al., 2020). At lower frequency, the peaks appeared at 814, 747, and 661 cm^−1^ are associated with the O–H and C–H vibrations in the ligand (Ivanchikova et al., 2014). In addition, a distinct peak occurred at 480 cm^−1^ is assigned to asymmetric stretching of Zr-(OC) (Wang et al., 2021a). Concerning CoFe_2_O_4_, two characteristic peaks are observed at 466 and 598 cm^−1^, which are related to the metal-oxygen stretching vibrations at the octahedral and tetrahedral sites in the spinel structure, respectively (Shahjuee et al., 2017). The other peaks at 1,065, 1,392, and 1,629 cm^−1^ are appeared by O–H, C–O, and C–H bending vibration, respectively (Yavari et al., 2016). Meanwhile, a weak band noted at 2,374 cm^−1^ might be resulted from C–H stretching vibration (Johnson et al., 2020). As for CoFe_2_O_4_@UiO-66, the characteristic spectral bands of UiO-66 can be observed, nevertheless, with less intensity and slight blue-shift, indicating the changing of the chemical environment around UiO-66 following the incorporation of CoFe_2_O_4_. Unlike UiO-66, a new peak can be identified around 500–600 cm^−1^ in the composite material that is associated with the stretching vibration of Fe–O band (Deng et al., 2013). This observation confirms the effective integration of UiO-66 and CoFe_2_O_4_ to form the composite material, which corresponds with the above XRD findings.

#### 3.1.2 Morphology analysis

**FIGURE 3:**
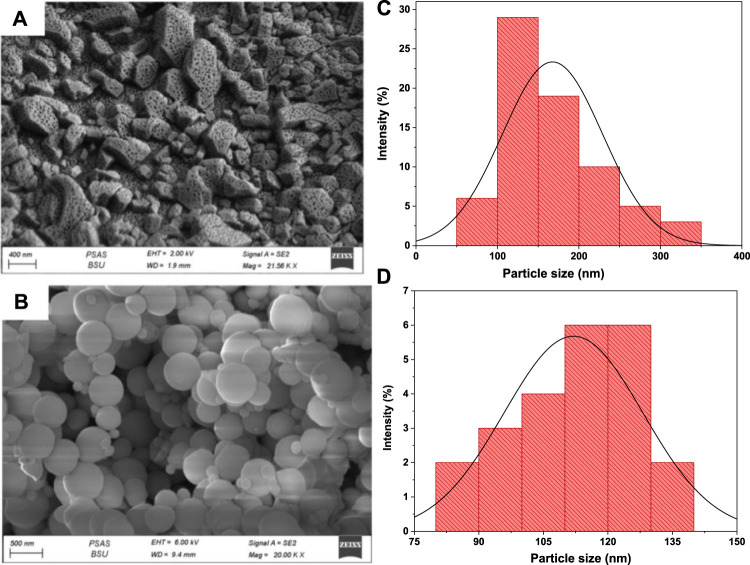
FESEM images of UiO-66 **(A)** and CoFe_2_O_4_@UiO-66 composite **(B)**; UiO-66 particle size distribution of UiO-66 **(C)** and CoFe_2_O_4_@UiO-66 composite **(D)**.

FESEM analysis was adopted to explore the morphological characteristics of bare UiO-66 and CoFe2O4@UiO-66 composite. As displayed in Figure 3A, UiO-66 exhibited irregular cubic morphology with an average diameter approaching between 150–200 nm (Figure 3C). In fact, with increasing the concentration of monocarboxylic acid modulators, more comparatively uniform pores are created in the MOF network (Wang et al., 2021b). This explains the porous surface of the as-synthesized UiO-66, which could be resulting from the high concentration of acetic acid modulator utilized during the preparation process. Figure 3B shows the FESEM image of the CoFe_2_O_4_@UiO-66 composite. It is interestingly noted that after coating with UiO-66, CoFe_2_O_4_ maintained the original spherical-like structure previously reported (Mu et al., 2021), with a relatively uniform size and rough surface. On the other hand, the crystal size of UiO-66 has reduced to ∼120 nm in the composite (Figure 3D), along with a morphological change from cubic to sphere-like crystals owing to the fast reaction between Zr and ligand (Han et al., 2017; Winarta et al., 2019).

**FIGURE 4:**
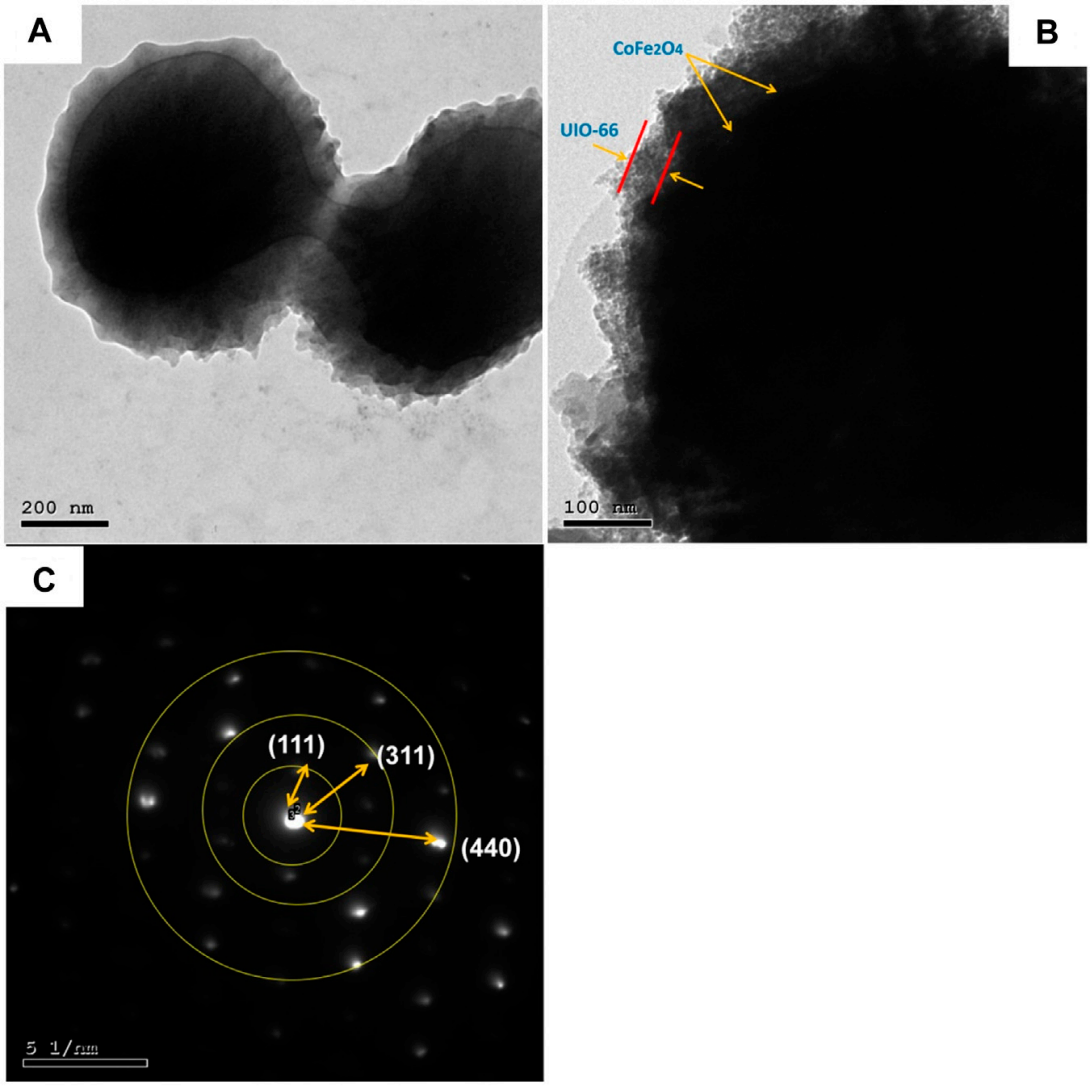
TEM images **(A,B)** and SAED pattern **(C)** of CoFe_2_O_4_@UiO-66 composite.

**FIGURE 5:**
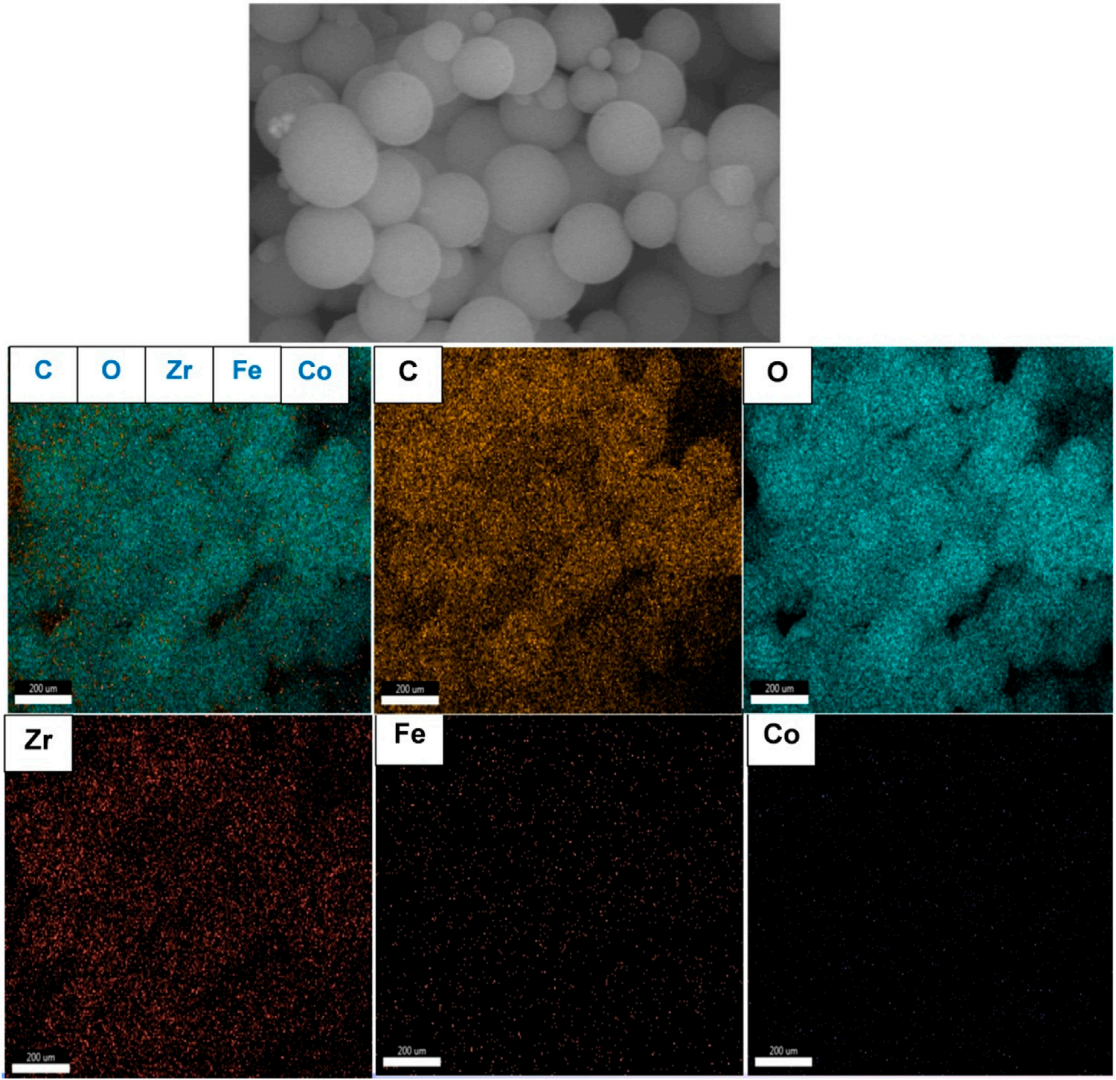
SEM-EDS elemental mapping of CoFe_2_O_4_@UiO-66 composite.

TEM study was further carried out to explore the microstructure of the as-synthesized composite. As presented in Figures 4A, B, CoFe_2_O_4_@UiO-66 displayed a distinct core-shell structure comprised of CoFe_2_O_4_ core with an average diameter of approximately 550 nm, coated by an outer UiO-66 shell with a thickness of 42.4 ± 11.9 nm. Importantly, an obvious contact interface between UiO-66 and CoFe_2_O_4_ can be seen that accelerates the migration of charge carriers, thereby enhancing the photocatalytic performance. The selected area electron diffraction (SAED) pattern of the CoFe_2_O_4_@UiO-66 composite (Figure 4C) demonstrated the polycrystalline nature with d-spacing of 0.49, 0.26, and 0.15 nm correlated to (111), (311), and (440) planes of the magnetic CoFe_2_O_4_. The EDS elemental mapping of the CoFe_2_O_4_@UiO-66 composite is illustrated in Figure 5. Homogeneous distribution of Zr, O, C, Fe, and Co elements can be observed. This low content for Fe and Co elements might be ascribed to the entire coating of UiO-66 on CoFe_2_O_4_ microspheres. In addition, the photograph of different samples further proved that pristine and composite materials were successfully fabricated (Supplementry Figure S2).

#### 3.1.3 XPS analysis

**FIGURE 6:**
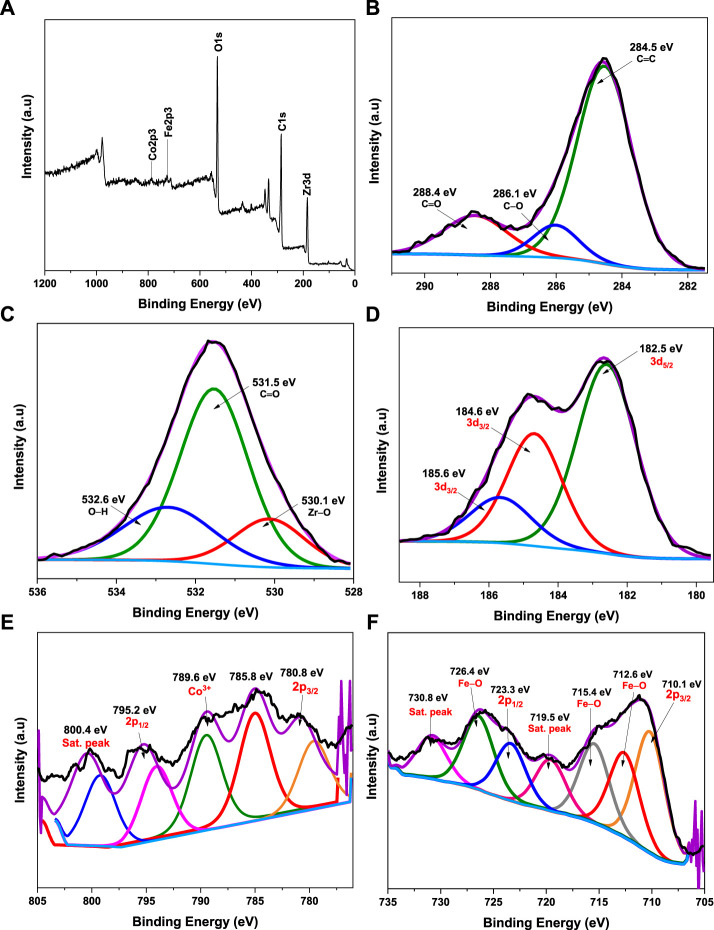
XPS spectra of CoFe_2_O_4_@UiO-66 composite: survey scan **(A)**, C 1s **(B)**, O 1s **(C)**, Zr 3 days **(D)**, Co 2p **(E)**, and Fe 2p **(F)**.

To explore the surface chemical state of the CoFe_2_O_4_@UiO-66 composite, the XPS spectra were recorded (Pan et al., 2022). The survey spectrum (Figure 6A) showed characteristic peaks for Zr 3d, Fe 2p, Co 2p, C 1s, and O 1s, accompanying intense peaks for the C and O elements corresponding to their relative abundance. For the C 1s spectrum (Figure 6B), three peaks at 284.5, 286.1, and 288.4 eV are respectively ascribed to C=C of the benzene ring and carboxylate groups of the BDC linker in the UiO-66 framework (Cao et al., 2018). As shown in Figure 6C, the O 1s spectrum demonstrated three deconvolution peaks cited at 530.1, 531.5, and 532.6 eV associated with the metal-oxygen bond (Zr–O) bond, C=O of the BDC linker, and surface adsorbed hydroxyl group, respectively (Yang et al., 2019). In Zr 3d XPS (Figure 6D), the characteristic binding energies of Zr 3d_5/2_ (at 182.5 eV) and Zr 3d_3/2_ (at 184.6 and 185.6 eV) can be seen, belonging to Zr–O core level interactions (Subhan et al., 2021). The energy spectrum of Co 2p depicted in Figure 6E demonstrated a pair of fitting peaks at 780.8 and 785.8 eV associated with Co 2p_3/2_ and another peak cited at 795.2 eV (with a relatively strong shake-up satellite peak at 800.4 eV) related to Co 2p_1/2,_ confirming the existence of Co^2+^ oxidation state in the spinal structure (Zhou et al., 2014; Chen et al., 2016). Typically, the satellite energy separation in oxides for Co^3+^ is approximately 8.5–9.5 eV (Xu et al., 2019). Given this, an extra peak centered at 789.6 eV corresponding to the binding energy of Co^3+^ 2p_3/2_ is presumably due to the surface oxidation of Co species after coating by UiO-66 particles. In the Fe 2p spectrum (Figure 6F), two distinct peaks located at 710.1 and 723.3 eV associated with the binding energies of Fe 2p_3/2_ and Fe 2p_1/2_, respectively, suggesting the existence of Fe^2+^ (Zhou et al., 2022), whereas, the Fe 2p shakeup satellites observed at 719.5 and 730.8 eV are assigned to Fe^3+^ spin state (Ma et al., 2015; Salunkhe et al., 2015). Moreover, the peaks noticed at 712.6, 715.4, and 726.4 eV could be due to Fe–O bonds, which further assert the strong interaction between CoFe_2_O_4_ and UiO-66 *via* Fe-O-Zr linkages (Xu et al., 2017).

#### 3.1.4 Surface area and thermal investigation

**FIGURE 7:**
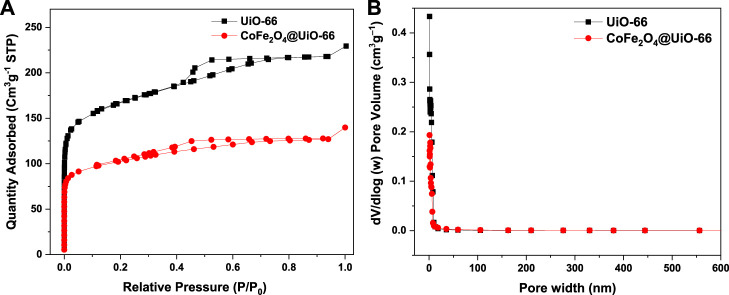
N_2_ adsorption-desorption isotherms **(A)**, and pore size distribution curves **(B)** of UiO-66 and CoFe_2_O_4_@UiO-66 composite.

N_2_ adsorption-desorption isotherm studies were employed to investigate the textural properties of UiO-66 and CoFe_2_O_4_@UiO-66 composite and relevant data are given in Figure 7; Table 1. UiO-66 (Figure 7A) displayed type IV isotherm with a well-defined H2 hysteresis loop, indicating the existence of mesopores, thereby authenticating the results of FE-SEM. The S_BET_ of UiO-66 is 593.94 m^2^ g^−1^. Obviously, the CoFe_2_O_4_@UiO-66 composite attained the same isotherm pattern; however, with an H4 type hysteresis loop and S_BET_ of 375.7 m^2^ g^−1^. The pore size distribution of as-synthesized catalysts also showed a similar trend (Figure 7B), whereas, the total pore volumes for UiO-66 and CoFe_2_O_4_@UiO-66 are calculated to be 0.35 and 0.21 cm^3^ g^−1^, respectively. Distinctly, this reduction in the S_BET_ (∼36%) and pore volume (∼40%) of the composite material is possibly connected with the formation of larger mesopores owing to the encapsulation of CoFe_2_O_4_ particles into the MOF network (Lee et al., 2015; Qi et al., 2019).

**TABLE 1:** Porous texture of the as-synthesized samples.

Sample	S_BET_ (m^2^ g^−1^)^a^	S_Langmuir_ (m^2^ g^−1^)^b^	V_t_ (cm^3^ g^−1^)^c^	Pore diameter (nm)^d^
UiO-66	593.94	742.31	0.35	2.36
CoFe_2_O_4_@UiO-66	375.7	480.03	0.21	2.27

^a^ BET specific surface area.

^b^ Langmuir specific surface area.

^c^ Total pore volume measured at P/P_0_ = .99.

^d^ Pore size in diameter calculated by the desorption data using Barrett–Joyner–Halenda (BJH) method.

**FIGURE 8:**
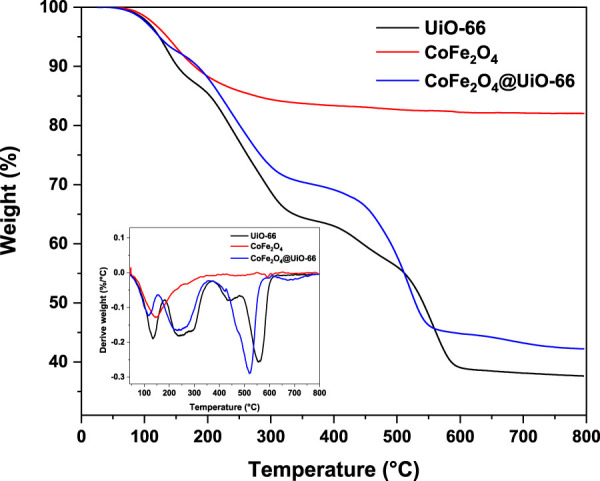
Thermal analysis of UiO-66, CoFe_2_O_4,_ and CoFe_2_O_4_@UiO-66 composite, inset: the corresponding derivative thermogravimetric (DTG) plots.

To evaluate the thermal behavior of the as-prepared catalysts, TGA analysis was conducted and the findings are displayed in Figure 8. For CoFe_2_O_4,_ two stages of weight loss with a total weight loss of ∼16% can be observed. The major one occurred up to 100°C owing to the evaporation of moisture, whilst, the minor mass loss happened at 330°C could be assigned to the elimination of ammonium hydroxide and chloride from the surface (Chakhtouna et al., 2021). In contrast, no considerable weight loss can be detected above 330°C, revealing the high thermal stability of CoFe_2_O_4_. On the other side, both pristine and modified UiO-66 demonstrated similar TGA curves with three stages of weight loss. In the case of pristine UiO-66, an initial weight loss (13%) occurs from 33°C–161°C owing to the evaporation of physically adsorbed water molecules from the UiO-66 surface (Xu et al., 2022). In the second stage, nearly 19% weight loss observed in the range of 161°C–300°C is associated with the removal of DMF molecules trapped inside the framework pores and thermal dehydration of zirconium clusters (Zhang et al., 2021a). The last weight loss (>30%) appeared at 430°C–600°C is ascribed to the thermal decomposition of the organic ligand to CO, CO_2,_ and ZrO_2_ (Molavi et al., 2018). Compared to bare UiO-66, the TGA curve of CoFe_2_O_4_@UiO-66 showed a relative reduction in weight loss by about 6.22%, indicating an improvement of thermal stability after the introduction of CoFe_2_O_4_ nanoparticles.

### 3.2 Optical properties

**FIGURE 9:**
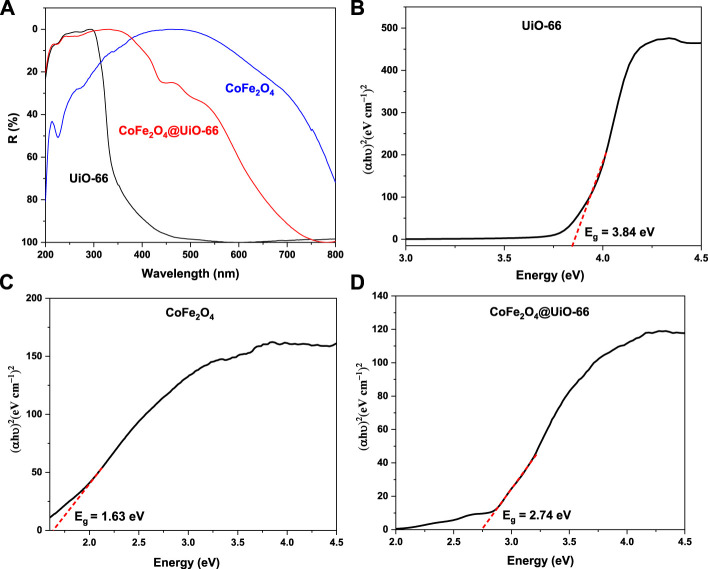
UV-vis DRS spectra **(A)**, bandgap (E_g_) plots **(B–D)** of the as-synthesized samples.

The UV-Vis DRS was performed to study the photoabsorption characteristics of the as-synthesized photocatalysts and findings are shown in Figure 9A. It can be noticed that bare UiO-66 displayed no absorption in the visible region, however, strong absorption is obvious in the UV spectral region with an absorption peak at 296 nm that can be ascribed to Zr–Oxo-clusters (Wang et al., 2016). Otherwise, owing to the black color of pristine CoFe_2_O_4_, absorption peaks can be seen in both UV and visible regions (Jing et al., 2016). By comparison, the light absorption edge of CoFe_2_O_4_@UiO-66 composite is red-shifted to around 467 nm, evidencing the enhancement of light absorption intensity and visible light utilization efficiency after combining CoFe_2_O_4_. For certifying, the Kubelka-Munk equation was applied to estimate the bandgap energy (E_g_) of semiconductors (Qiu et al., 2019):

$${\left(\alpha h\upsilon \right)}^{2}\;=\;A{\left(h\upsilon –E_{g}\right)}^{n/2}$$ (2)

where α, h, υ, and A are the diffuse absorption coefficient, Planck’s constant, light frequency, and constant, respectively. As depicted in Figures 9B–D, direct bandgap energies were calculated from the tangent line obtained by plotting (αhυ)^2^ vs energy (hυ). For UiO-66, CoFe_2_O_4_, and CoFe_2_O_4_@UiO-66 composite, the estimated E_g_ values are approximately 3.84, 1.63, and 2.74 eV, respectively. In the case of the composite, the relatively reduced bandgap observed might be assigned to the interface formed between UiO-66 and the narrow bandgap CoFe_2_O_4_ particles, resulting in more effective absorption of the solar spectrum and eventually better photocatalytic response.

### 3.3 Photoelectrochemical properties

**FIGURE 10:**
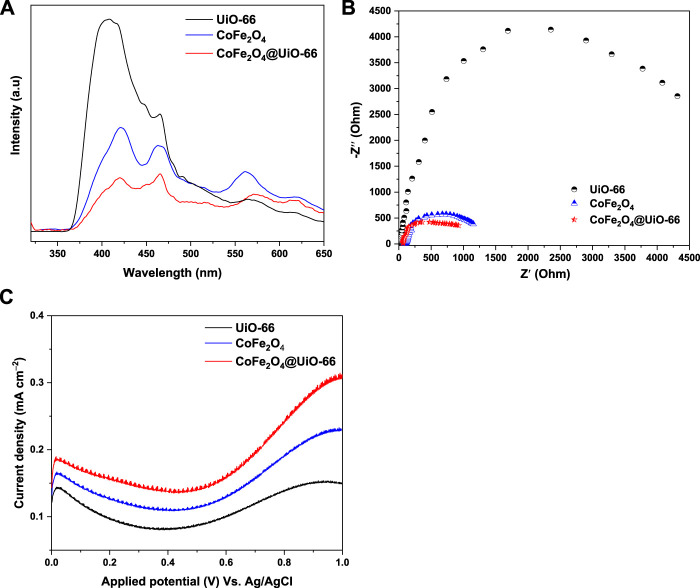
PL emission spectra **(A)**, EIS Nyquist **(B)**, and LSV curves **(C)** of UiO-66, CoFe_2_O_4_ and CoFe_2_O_4_@UiO-66 composite.

Photoluminescence (PL) spectra were obtained to evaluate charge separation and transmission efficiency over different catalysts. In theory, the lower the PL intensity, the lower the reintegration of the charge carriers, which is advantageous to the photocatalytic reaction (He et al., 2021). As revealed in Figure 10A, the PL spectral intensity decreased in the order of UiO-66 > CoFe_2_O_4_ > CoFe_2_O_4_@UiO-66, where, pristine UiO-66 exhibited the highest peak intensity at around 407 nm. Conversely, upon coupling with CoFe_2_O_4_ particles, the PL intensity is markedly suppressed and the signal is displaced to a higher wavelength (466 nm). Thus, it can be deduced that the formation of core-shell heterostructure significantly quenched the recombination of photoinduced charge carriers, accelerating the migration rate.

The photoelectrochemical characterizations were further investigated by electrochemical impedance spectroscopy (EIS) and linear sweep voltammetry (LSV) to verify the charge transfer and current density, thereby determining the photocatalytic performance of catalysts. Consistent with the findings of the PL analysis, EIS Nyquist plots displayed the same tendency (Figure 10B). Basically, the smaller the semicircle diameter in EIS plots, the lower the charge transfer resistance (Zhang et al., 2022). In this study, it is interestingly noted that CoFe_2_O_4_@UiO-66 displayed a smaller Nyquist arc radius than those of parent UiO-66 and CoFe_2_O_4_, reaffirming the depletion in charge transfer resistance and enhancement of charge carriers separation by constructing heterojunction. In addition, LSV profiles of as-synthesized catalysts are represented in Figure 10C. As can be seen, UiO-66 displayed the lowest current density (0.15 mA cm^−2^), owing to inefficient utilization of visible light. In contrast, the current density is significantly improved to 0.31 mA cm^−2^ over CoFe_2_O_4_@UiO-66. On the other side, the anodic currents in LSV curves demonstrated the n-type semiconductor nature of the as-synthesized catalysts (Quach et al., 2022). To summarize, these findings assert that the successful interfacial contact between UiO-66 and CoFe_2_O_4_ can sufficiently hinder the charge recombination dilemma and induce effective separation of photoinduced carriers, leading to swift surface reaction dynamics and better photocatalytic activity.

### 3.4 Photocatalytic performance

**FIGURE 11:**
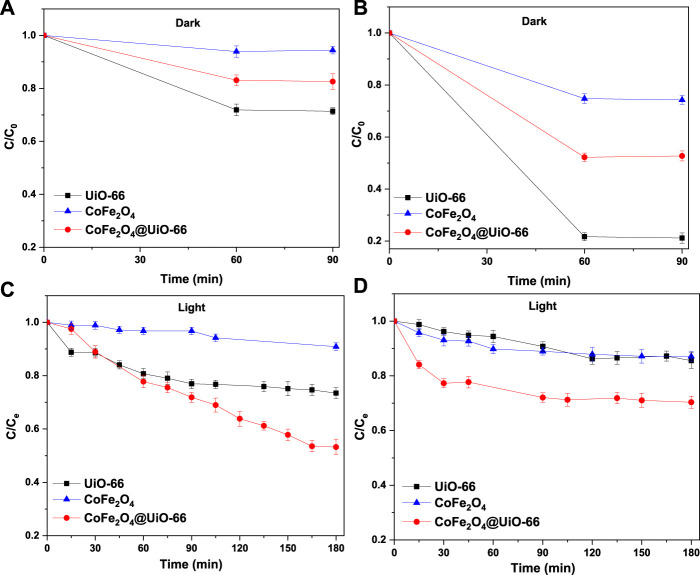
Adsorption and photodegradation performance of as-synthesized photocatalysts for removal of MB **(A,C)** and MO **(B,D)** dyes.

**FIGURE 12:**
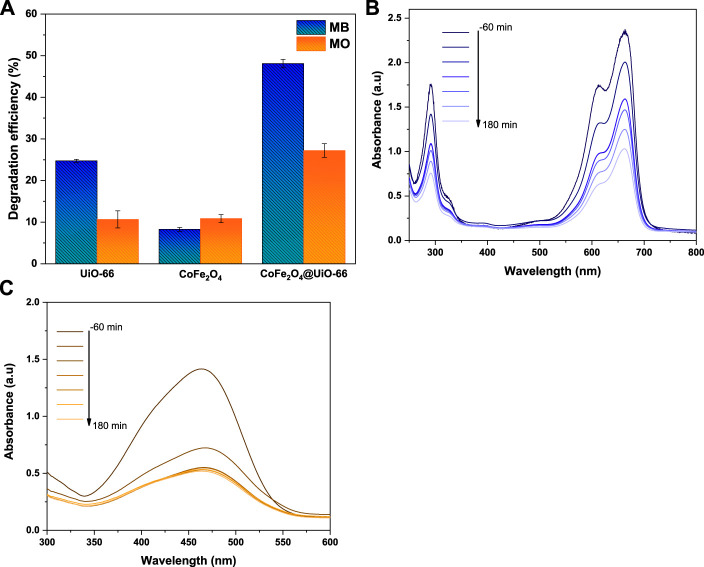
The photodegradation efficiency of as-synthesized photocatalysts **(A)**, UV-Vis absorption spectra for degradation of MB **(B)** and MO **(C)** dyes over CoFe_2_O_4_@UiO-66.

The photocatalytic activities of bare and composite catalysts were studied through the degradation of MB and MO dyes as representative pollutants under simulated solar irradiation. Obviously, under dark conditions, the dye adsorption capacity follows the order UiO-66 > CoFe_2_O_4_@UiO-66 > CoFe_2_O_4_ (Figures 11A, B). In comparison with pure CoFe_2_O_4_, the adsorption capacity of CoFe_2_O_4_@UiO-66 composite is significantly boosted owing to the abundant exposed adsorption sites and internal porous structure of the outer UiO-66 shell. Noteworthy, the removal efficiencies of the different photocatalysts for MO dye are about 3.0–4.4-folds higher than that for MB dye. In general, the surface charge of the particles can prominently influence their interaction with target pollutants, affecting the adsorption capacity (Sohrabnezhad and Moghadamy, 2022). In this regard, the Zeta potentials of the as-synthesized catalysts were measured and the findings are depicted in (Supplementary Figure S3). As observed, all samples possess positive charges with a potential of +17.2, + 19.5, and +23.8 mV for UiO-66, CoFe_2_O_4,_ and CoFe_2_O_4_@UiO-66, respectively. Hence, anionic dye molecules are effectively adsorbed to the surface by electrostatic interaction, which is proposed as the predominated removal mechanism during the adsorption process. Following the accomplishment of adsorption-desorption equilibrium, the equilibrium dye concentration (C_e_) was applied as the initial concentration. As presented in Figures 11C, D, upon simulated solar illumination, pristine UiO-66 and CoFe_2_O_4_ exhibited low degradation efficiency due to the weak visible light harvesting and ineffective segregation of photogenerated carriers, respectively. By comparison, considerable photocatalytic degradation was attained in presence of CoFe_2_O_4_@UiO-66 composite, with a 20%–40% increase in degradation efficiency (Figure 12A). The UV-Vis spectral changes of MB and MO dyes over CoFe_2_O_4_@UiO-66 photocatalyst at different illumination times are revealed in Figures 12B, C.

To further elucidate the photodegradation process, kinetic curves were plotted and rate constants k) were calculated. (Supplementary Figure S4) illustrates the pseudo-first-order kinetic equation to define the reaction rate constant of different samples following Eq. 3 (Yi et al., 2019):

$$(\ln\left(C_{e}/C\right)=kt$$ (3)

where k is the first-order rate coefficient (min^−1^), C_e_ is the dye concentration at equilibrium and C is the concentration at time t. It is noteworthy that among the three catalysts, the CoFe_2_O_4_@UiO-66 composite possessed a greater rate constant, which is consistent with the photocatalytic results. As evident, the heterojunction constructed between UiO-66 and CoFe_2_O_4_ simultaneously reduced the charge carrier recombination and increased the photon absorption capacity, resulting in a faster photocatalytic reaction. The photocatalytic activity of CoFe_2_O_4_@UiO-66 composite for dye degradation was further compared with the previously reported photocatalysts (Table 2). Overall, the CoFe_2_O_4_@UiO-66 composite displayed outstanding efficiency for the degradation of dyes at high initial concentrations.

**TABLE 2:** Comparison of photocatalytic performance of CoFe_2_O_4_@UiO-66 with other reported photocatalysts for degradation of MB and MO.

Photocatalyst	Dye concentration (mg/L)	Catalyst amount (mg/L)	Light source	Irradiation time (min)	Removal rate (%)	Ref
MB dye						
Fe-UiO-66	20	10	60 W white LED lamp	160	84	Hosseini et al. (2022)
g-C_3_N_4_−xClx/0.5 M HCl	3	25	1000 W Xe lamp (λ ≥ 420 nm)	180	97	Bai et al. (2020)
Co_0.1_Mg_0.9_Fe_2_O_4_	10	10	A halogen lamp (intensity: 70 mWcm¯^2^)	240	80	Dojcinovic et al. (2021)
*α*-Fe_2_O_3_@UiO-66	13	100	300 W Xe lamp (λ ≥ 420 nm)	50	100	Zhang et al. (2019a)
UiO-66/g-C_3_N_4_ UC10:10	10	50	350 W Xe lamp (λ > 420 nm)	240	99	Zhang et al. (2018)
30% CuNb_2_O_6_/g-C_3_N_4_	10	20	500 W Xe lamp	150	98.5	Ahmad et al. (2022)
S–N-co-doped-CoFe_2_O_4_@rGO@TiO_2_	5	8	300 W Xe lamp (λ > 420 nm)	360	94	Wei et al. (2019)
CoFe_2_O_4_@UiO-66	100	50	150 W Xe lamp	180	56.7	This work
MO dye						
UiO-66-NH_2_@CNT (3 wt%)	15	30	100 W LED lamp	30	93	Abdi et al. (2021)
OV-BOC	10	100	300 W Xe lamp (λ > 400 nm)	120	82	Zhao et al. (2019)
40 wt%-AgBr/CeO_2_	30	50	300 W Xe lamp (λ ≥ 400 nm)	180	93	Chen et al. (2021)
UiO-66/BiFeO_3_	10	50	250 W high-pressure Hg lamp (λ > 400 nm)	180	88.7	Bargozideh et al. (2020)
Au-CoFe_2_O_4_/MoS_2_	50	70	300 W iodine tungsten lamp	120	99	Jia et al. (2019)
rGO@In_2_S_3_@UiO-66	15	30	500 W Xe lamp (λ = 420 nm)	60	98.1	Gan et al. (2019)
3% TiO_2_/g-C_3_N_4_	10	1,000	500 W Xe lamp (simulated sunlight)	80	62.6	Huang et al. (2016)
CoFe_2_O_4_@UiO-66	100	50	150 W Xe lamp	180	63.3	This work

### 3.5 Proposed photocatalytic mechanism

**FIGURE 13:**
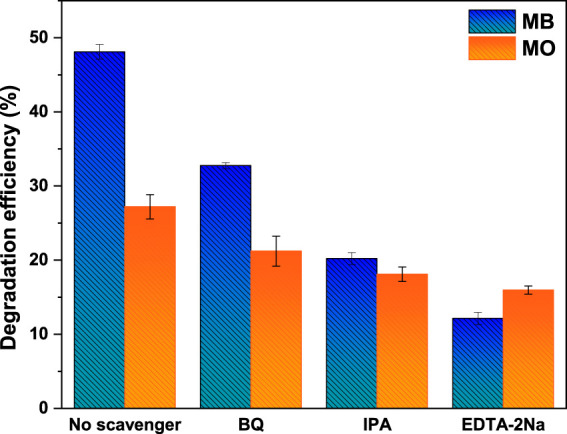
The effect of different quenchers on the photocatalytic activity of CoFe_2_O_4_@UiO-66 for degradation of MB and MO dyes.

To explore the possible mechanism for the photocatalytic degradation of MB and MO dyes, radical trapping experiments were performed over CoFe_2_O_4_@UiO-66 composite under simulated solar irradiation. EDTA-2Na, BQ, and IPA were utilized separately in the degradation system as h^+^, •O_2_^−^, and •OH scavengers, respectively. As observed in Figure 13, all the reactive substances are contributed to the catalytic process. Nevertheless, EDTA-2Na has the most significant impact on the degradation reaction. When EDTA-2Na is added, the degradation rate decreased drastically to 12% and 15% for MB and MO, respectively. In the meanwhile, upon the introduction of BQ and IPA, a moderate influence on the degradation efficiency can be seen. This implies that photogenerated holes are the dominant active species, while •O_2_^−^ and •OH possess a certain contribution to the photocatalytic reaction.

**FIGURE 14:**
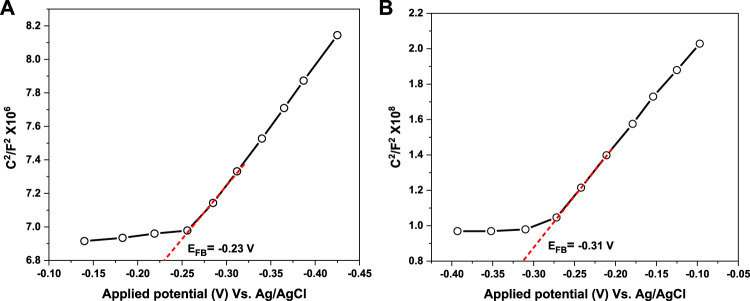
Mott-Schottky plots of UiO-66 **(A)** and CoFe_2_O_4_ **(B)**.

For more insights into the mechanism of photogenerated charge separation, Mott-Schottky (M−S) measurement was further implemented to investigate the electronic band structure and the semiconductivity nature of UiO-66 and CoFe_2_O_4_. The positive slope of the tangent lines depicted in Figures 14A, B suggests that both materials are typical n-type semiconductors (Shen et al., 2015a), which is in agreement with the LSV results. The results showed that the flat band potential (E_FB_) of UiO-66 and CoFe_2_O_4_ are set as –0.23 and –0.31 V vs. Ag/AgCl, respectively. Subsequently, the E_FB_ (vs. NHE) could be determined as follows (Man et al., 2022):

$$E_{\left(NHE,\;pH=7\right)}=E_{Ag/AgCl}–0.059\left(7–pH\;of\;the\;electrolyte\right)+0.198$$ (4)

Thence, the E_FB_ of UiO-66 and CoFe_2_O_4_ is –0.24 and –0.36 V (vs NHE), respectively. In general, the conduction band (E_CB_) potential for n-type semiconductors is approximately 0.1–0.2 V more negative than the flat band potential (Ishikawa et al., 2002). Accordingly, the corresponding (E_CB_) potential of UiO-66 and CoFe_2_O_4_ can be calculated as –0.44 and –0.56 V (vs. NHE), respectively. From the bandgap values obtained above, the valence band (E_VB_) potential can be calculated using Eq. 4:

$$E_{VB}=E_{g}+E_{CB}$$ (5)

Subsequently, the E_VB_ potential of UiO-66 and CoFe_2_O_4_ is determined as 3.40 and 1.07 V (vs. NHE), respectively.

**FIGURE 15:**
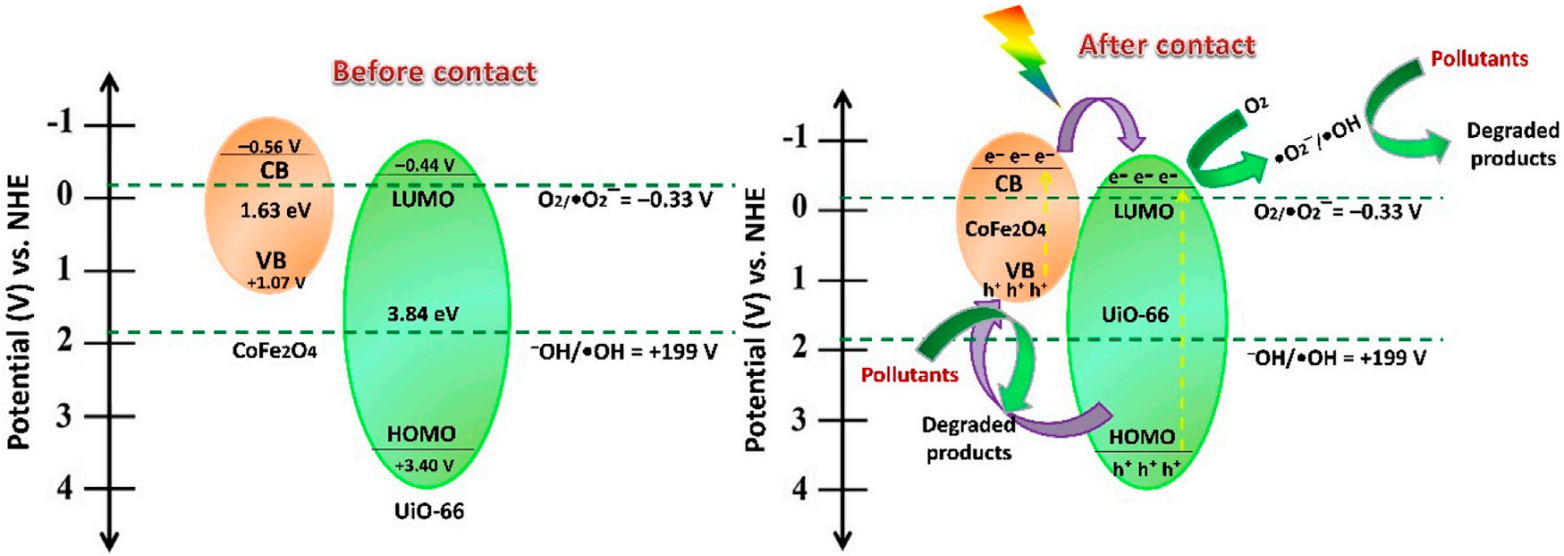
A proposed photocatalytic mechanism for dye degradation over CoFe_2_O_4_@UiO-66 heterojunction under simulative solar irradiation.

In the light of the aforementioned findings and discussion, the plausible reaction mechanism for photocatalytic degradation of MB and MO over CoFe_2_O_4_@UiO-66 photocatalyst is proposed (Figure 15). Upon simulated sunlight irradiation, both UiO-66 and CoFe_2_O_4_ are excited, generating electrons (e^–^) and holes (h^+^) in their CB and VB, respectively. Since, the CB potential of CoFe_2_O_4_ (–0.56 V) is more negative than the LUMO of UiO-66 (−0.44 V), the excited electrons can directly transfer through the interface channels formed by the heterojunction to the LUMO of UiO-66, suppressing the recombination of photogenerated carriers (Wang et al., 2021d; Gao et al., 2021). Subsequently, the photoinduced electrons at LUMO of UiO-66 can reduce the dissolved oxygen to yield •O_2_^−^ radicals. Meanwhile, the photogenerated holes would transfer from the VB of UiO-66 (+3.40 V) to the VB of CoFe_2_O_4_ (+1.07 V). However, as the VB potential of CoFe_2_O_4_ (+1.07 V) is lower than the redox potential of^–^OH/•OH (1.99 V vs NHE), the photogenerated holes cannot oxidize H_2_O to produce •OH radicals (Wang et al., 2022). Instead, the accumulated holes promptly degrade the dye molecules because of their strong oxidation properties. Otherwise, •OH reactive radicals could be indirectly generated through •O_2_^−^ radicals at the CB of the photocatalyst (Zou et al., 2021). This is consistent with the results of quenching experiments, indicating the construction of a staggered type II heterojunction energy band alignment near the interface of UiO-66 and CoFe_2_O_4_. The following equations may summarize the degradation process:

$${CoFe}_{2}O_{4}@UiO-66+h\upsilon \;\to \;e^{–}+h^{+}$$ (6)

$$e^{–}+O_{2}\;\to \;•O_{2}^{–}$$ (7)

$$•O_{2}^{–}+2h^{+}+2e^{–}\;\to \;H_{2}O_{2}$$ (8)

$$H_{2}O_{2}+2e^{–}\;\to^{-}OH+•OH$$ (9)

$$Organic\;dye+h^{+}+•OH+•O_{2}^{–}\;\to \;degradation\;products$$ (10)

## 4 Conclusion

In summary, a novel CoFe_2_O_4_@UiO-66 core-shell heterojunction photocatalyst was successfully synthesized through a simple solvothermal route. In comparison with the UV-driven UiO-66 catalyst, the CoFe_2_O_4_@UiO-66 heterojunction displayed an enhanced photo-responsive capacity in the visible region with an absorption band of ∼467 nm. This can be certified by the reduction of bandgap energy from 3.84 eV for UiO-66 to 2.74 eV for the composite material. The CoFe_2_O_4_@UiO-66 composite exhibited better performance than either UiO-66 or CoFe_2_O_4_ towards photodegradation of organic dyes at a high initial concentration under simulated solar light irradiation. The overall removal efficiency of dyes (100 mg/L) over CoFe_2_O_4_@UiO-66 (50 mg/L) reached >60% within 180 min irradiation. Moreover, the photoluminescence, impedance, and current density studies showed an effective charge separation and transfer over the CoFe_2_O_4_@UiO-66 composite. This was mainly ascribed to the tight interfacial contact formed through the heterojunction, which suppressed the charge recombination rate, thereby improving the photocatalytic activity. From radical scavenging experiments and Mott-Schottky analysis, it can be inferred that h^+^ had the primarily significant contribution during the photocatalytic process. This study paved the way to design MOF-based core-shell heterostructured photocatalysts with more active sites, good optical properties, and enhanced photocatalytic activity for various environmental applications.

## Data Availability

The original contributions presented in the study are included in the article/Supplementary Material, further inquiries can be directed to the corresponding author.
