# Supplementary material for: In-situ construction of Zr-based metal-organic framework core-shell heterostructure for photocatalytic degradation of organic pollutants
Source: Front Chem. 2023 Jan 4;10:1102920. doi: 10.3389/fchem.2022.1102920 (PMC9845943; doi:10.3389/fchem.2022.1102920)
Supplement: Supplementary file 1 [file DataSheet1.docx]

**Supporting information**

**In-situ construction of Zr-based metal-organic framework core-shell heterostructure for photocatalytic degradation of organic pollutants**

Yasmeen S. Abdel Aziz^1^, Moustafa M.S. Sanad^2^, Reda M. Abdelhameed^3^, Ayman H. Zaki^4,5*^

*^1^ National Institute of Oceanography and Fisheries (NIOF), Scopus affiliation ID 57218369725, Cairo 11562, Egypt*

*^2^ Central Metallurgical Research and Development Institute, (CMRDI) P.O. Box 87, Helwan, 11421, Cairo, Egypt*

*^3^ Applied Organic Chemistry Department, Chemical Industries Research Institute, National Research Centre, Scopus affiliation ID 60014618, 33 EL Buhouth St., Dokki, Giza 12622, Egypt*

*^4^ Materials Science and Nanotechnology Department, Faculty of Postgraduate Studies for Advanced Sciences, Beni-Suef University, Egypt*

*^5^ National Institute for Materials Science, Japan*

**Table S1**

**Physicochemical properties of dyes**

| Ionicity | pKa | Molecular weight (g/mol) | Chemical structure | Chemical formula | Organic dye |
| --- | --- | --- | --- | --- | --- |
| Cationic | 3.14 | 319.85 | 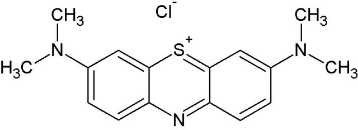 | C_16_H_18_ClN_3_S | Methylene blue |
| Anionic | 3.58 | 327.33 | **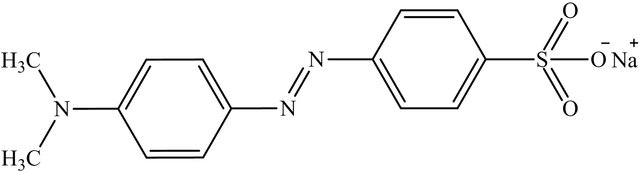** | C_14_H_14_N_3_NaO_3_S | Methyl orange |

**Fig. S1.** Simulated XRD patterns of UiO-66 (a) and CoFe_2_O_4_ (b).


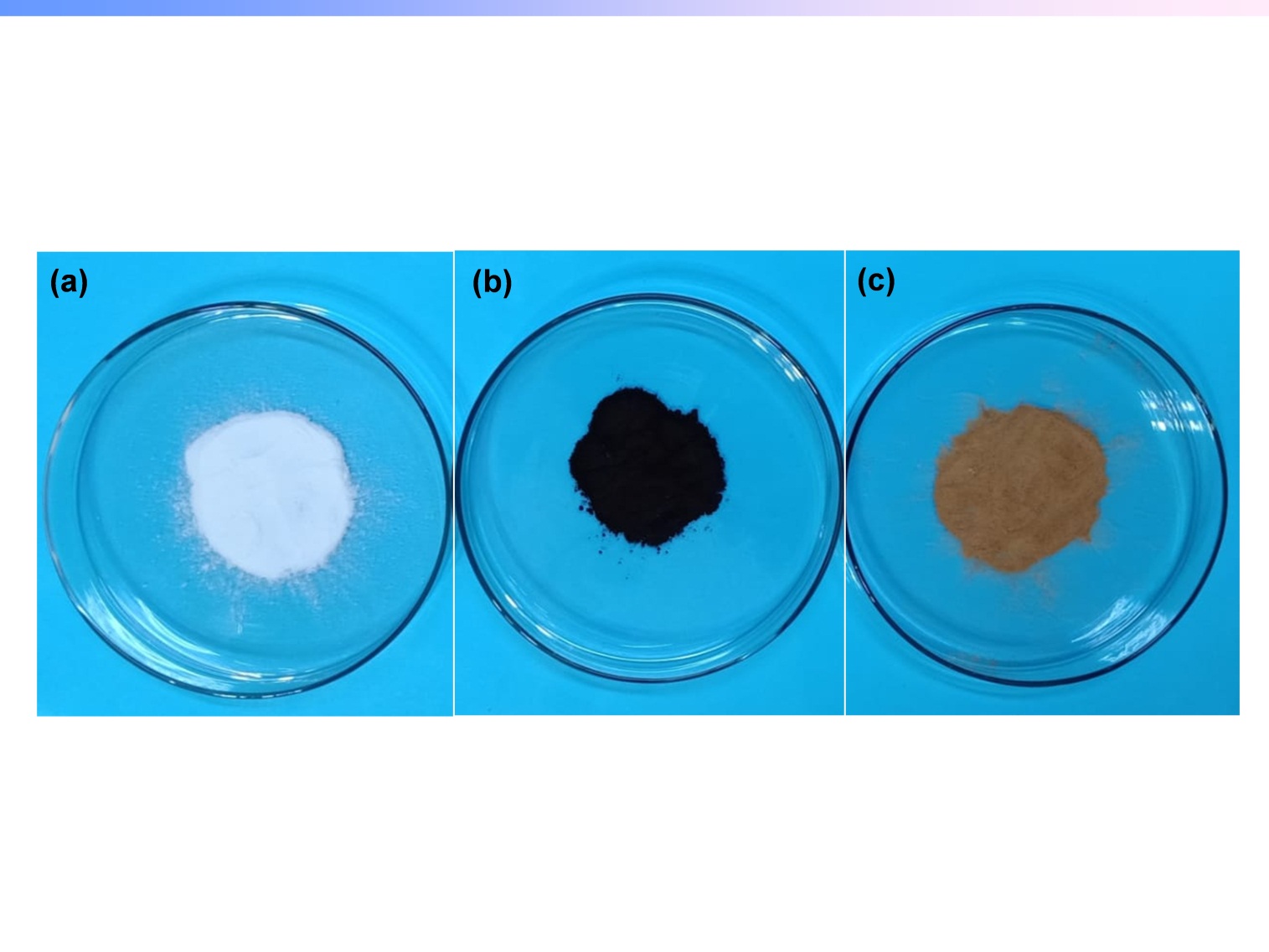


**Fig. S2.** Photographs of (a) UiO-66, (b) CoFe_2_O_4_ and (c) CoFe_2_O_4_@UiO-66 composite.

**Fig. S3.** Zeta potential of UiO-66 (a), CoFe_2_O_4_ (b), and CoFe_2_O_4_@UiO-66 composite (c).

**Fig. S4.** Kinetic plots for photodegradation of MB (a) and MO (b) dyes over various photocatalysts under simulated solar irradiation, inset: the corresponding reaction rate constants (k values).
